# Assessing the Effect of the Refresh Rate of a Device on Various Motion Stimulation Frequencies Based on Steady-State Motion Visual Evoked Potentials

**DOI:** 10.3389/fnins.2021.757679

**Published:** 2022-01-07

**Authors:** Chengcheng Han, Guanghua Xu, Xiaowei Zheng, Peiyuan Tian, Kai Zhang, Wenqiang Yan, Yaguang Jia, Xiaobi Chen

**Affiliations:** ^1^School of Mechanical Engineering, Xi’an Jiaotong University, Xi’an, China; ^2^State Key Laboratory for Manufacturing System Engineering, Xi’an Jiaotong University, Xi’an, China

**Keywords:** motion perception, refresh rate, brain computer interface (BCI), steady-state motion visual evoked potential (SSMVEP), electroencephalogram (EEG)

## Abstract

The refresh rate is one of the important parameters of visual presentation devices, and assessing the effect of the refresh rate of a device on motion perception has always been an important direction in the field of visual research. This study examined the effect of the refresh rate of a device on the motion perception response at different stimulation frequencies and provided an objective visual electrophysiological assessment method for the correct selection of display parameters in a visual perception experiment. In this study, a flicker-free steady-state motion visual stimulation with continuous scanning frequency and different forms (sinusoidal or triangular) was presented on a low-latency LCD monitor at different refresh rates. Seventeen participants were asked to observe the visual stimulation without head movement or eye movement, and the effect of the refresh rate was assessed by analyzing the changes in the intensity of their visual evoked potentials. The results demonstrated that an increased refresh rate significantly improved the intensity of motion visual evoked potentials at stimulation frequency ranges of 7–28 Hz, and there was a significant interaction between the refresh rate and motion frequency. Furthermore, the increased refresh rate also had the potential to enhance the ability to perceive similar motion. Therefore, we recommended using a refresh rate of at least 120 Hz in motion visual perception experiments to ensure a better stimulation effect. If the motion frequency or velocity is high, a refresh rate of≥240 Hz is also recommended.

## Introduction

An accurate presentation of stimuli is a prerequisite for accurate results of visual perception experiments. However, most modern monitors present motion objects at discrete locations, showing an approximate continuous motion process (Chapiro et al., 2019). Motion blur will occur when the motion speed or motion frequency is too high; it violates the assumption of smooth motion and causes stimulus distortion and false experimental results (Tourancheau et al., 2009; Watson and Ahumada, 2011). Since motion blur is positively related to speed and inversely related to the refresh rate of a device, increasing the refresh rate of the monitor used in experiments has become a mainstream choice in visual motion perception research. However, hardware cost and software compatibility limit the maximum refresh rate that can be employed. Therefore, it is an important part of visual motion perception research to analyze the effect of the monitor refresh rate and select the monitor with an appropriate refresh rate according to the stimulus.

Many studies have been conducted on the effect of the refresh rate of a device on visual motion perception with psychological methods. For example, Mulholland et al. (2015) used the contrast thresholds method to evaluate the effect of the refresh rate on temporal summation, and a CRT (cathode-tube-ray) monitor with refresh rates of 60 and 160 Hz was used as the visual stimulation device. Kime et al. (2016) evaluated perceptual performance with a digital micromirror device (DMD) with a high refresh rate of 1,000 Hz and a normal refresh rate of 60 Hz. Denes et al. (2020) asked participants to observe motion stimuli at different speeds (15, 30, and 45 deg/s) on liquid crystal displays (LCDs) with different refresh rates (50–165 Hz) and evaluated the display quality with participative just-noticeable differences (JND) indicators. However, due to differences in motion stimulus parameters or evaluation indicators, different studies have reached different conclusions on the effect of the refresh rate of a device on the improvement of visual motion perception. In particular, most reports use participative psychological methods or sampling theory to study the effect of the refresh rate of a device on visual motion perception (Kuroki et al., 2006, 2007; Noland, 2014); hence, it is time-consuming to conduct a continuous quantitative analysis of the interaction between the refresh rate of a device and motion frequency (or motion velocity) due the vast number of values possible for many parameters (Emoto et al., 2014). These problems limit refresh rate research in visual motion perception, and it is difficult to provide suitable suggestions for the selection of a refresh rate in visual experiments.

Brain computer interface (BCI) is a technology that directly converts brain activity into external instructions (Wolpaw et al., 2002; Nicolasalonso and Gomezgil, 2012), and many researchers have noticed the potential of BCI in visual perception and medical diagnosis (Norcia et al., 2015; Nakanishi et al., 2017; Overbeek et al., 2018; Guger et al., 2021). Among them, the steady-state visual evoked potential (SSVEP) (Middendorf et al., 2000) method uses visual stimuli of a specific frequency to induce steady-state potentials. The SSVEP method is a relatively mature electroencephalogram (EEG)-BCI technology (Guger et al., 2001; Bashashati et al., 2007), it has the characteristics of a high SNR (signal-noise ratio). Compared with the broadband distribution of noise signals, the SSVEP response has a narrow-band distribution. By defining a specific frequency, researchers can record the subtle differences of different visual stimuli responses in a short period of time. Moreover, researchers can measure the SSVEP response without noting obvious behavior, control the influence of decision criteria after the sensory or perceptual coding stage (de Lissa et al., 2020), and provide a quantitative method for visual perception research on refresh rates (Gembler et al., 2017; Nagel et al., 2018; Başaklar et al., 2019).

In addition to the commonly used flicker or pattern-reversal stimulation methods, motion stimulation can also elicit a steady-state response, which can be called steady-state motion visual evoked potentials (SSMVEPs) (Xie et al., 2011); In recent years, some researchers have tried to use the SSMVEP method to analyze the effect of the refresh rate of a device on visual motion perception. For example, Khoei et al. (2018) found that coherent trajectory SSMVEP stimuli (3 Hz) induced stronger responses at high refresh rates, and they suggested that a display with higher refresh rates (≥240 Hz) should be used to induce visual perception cortical responses. Chai et al. (2020) used the SSMVEP paradigm (8–15 Hz) to induce visual cortical responses with monitors that had refresh rates of 60 and 144 Hz. However, they reported that the refresh rate of the monitor had no significant effect on the improvement of the evoked response. These studies use flicker or size scaling as the stimulus targets, resulting in changes in brightness that interfere with the ability to achieve an accurate motion perception response. Moreover, the frequency range of the above SSMVEP experiment was limited, and the effect of the refresh rate of the monitor on high-frequency or high-speed motion was not analyzed. The design flaws of pattern and frequency in the SSMVEP paradigm made the research results incomprehensive.

The goal of this study is to offer an analysis method of the effect of the refresh rate of a device on visual motion perception using broadband flicker-free SSMVEP (Han et al., 2018). The flicker-free SSMVEP paradigm utilizes the contraction and expansion of the checkerboard texture, which has the characteristics of low flicker and concentrated spectral peaks; also, it is convenient for the analysis of response changes under different conditions. In this study, the frequency of the stimulus is set to 7–28 Hz, the motion form of the paradigm is modulated by sine waves and triangle waves, and the monitor refresh rates are 60, 120, and 240 Hz. By analyzing the difference in induced response intensity under different refresh rates, stimulation frequencies and motion forms, we comprehensively evaluate the effect of the refresh rate. Considering that the multiparameter experiment is time-consuming and easily induces visual fatigue, this study uses the sweep method to linearly modulate the stimulus frequency, quickly induce a continuous broadband visual response, and avoid interference from the evoked potential response.

## Materials and Methods

### Participants

Seventeen healthy participants (with normal or corrected-to-normal vision) participated in the experiment in this study (including 7 women; age 20–25 years, average age 22 years). Before the test, all experiment participants received training to familiarize themselves with the experimental process. All participants were asked to sign informed written consent following a protocol approved by the institutional review board of Xi’an Jiaotong University and that conformed to the Declaration of Helsinki.

### Environment and Data Acquisition

The visual stimulator was an ASUS PG258Q 24.5-inch LCD monitor (1,920 × 1,080 pixels, 543.7 × 302.6 mm, the actual width of each pixel was approximately 0.28 mm, and the maximum supportable refresh rate was 240 Hz). The experiment was carried out in a quiet room with general lighting. All participants were asked to sit in comfortable armchairs 65 cm in front of the LCD monitor.

The EEG signals were recorded with ZhenTec NT1 (ZhenTec Intelligence Ltd., China). The electrodes were arranged according to the international 10–20 electrode system. A total of 6 electrodes were arranged. These electrodes were placed in the occipital region (POz, PO3, PO4, Oz, O1, and O2), the reference channel was set in the unilateral earlobe (A1), and the ground channel was set in the middle of the forehead (Fpz). The acquisition device sampled EEG signals at a frequency of 1,200 Hz, the bandpass filter was set at 2–100 Hz, and the notch filter was set at 48–52 Hz. The impedance of all electrodes was kept below 5 kOhms.

### Paradigm Design and Experiment Process

Our paradigm design utilized motion checkerboard patterns to construct flicker-free SSMVEP visual stimuli paradigm (Han et al., 2018), motion checkerboard paradigm have the characteristics of low contrast and low visual fatigue (Xie et al., 2016; Zheng et al., 2019, 2020b), it can avoid the effects of fatigue on the response of evoked potential. The motion checkerboard pattern consisted of eight concentric rings, and each ring was divided into 24 equal sectors of black and white squares. In the experiment, participants were asked to gaze at motion stimuli without head movement or eye movement. In order to avoid interference from surrounding stimuli, the single-target stimulation paradigm was used. Since the evoked visual potential is most affected by the parameters in the visual field center stimulus, the experiment results of the paradigm can ensure the accuracy of the analysis conclusions.

**FIGURE 1:**
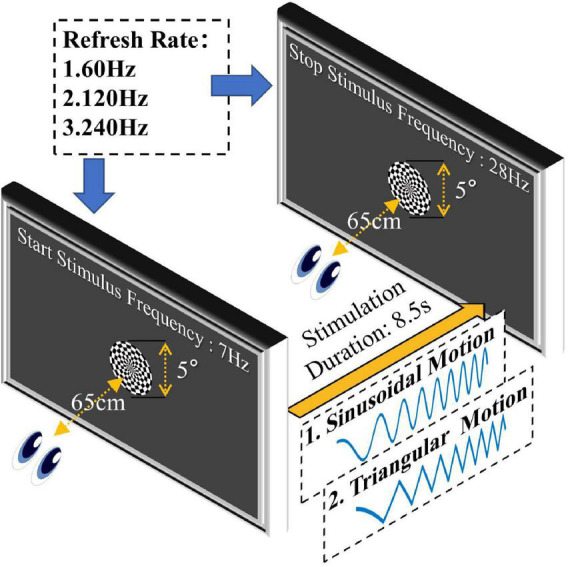
Stimulation parameter settings of the paradigm. The motion stimulus was modulated by sinusoidal or triangular sweep signals. The duration of stimulation was set at 8.5 s, the start frequency was set at 7 Hz, the end frequency was set at 28 Hz, the viewing angle of the stimulus was set at 5°, and the motion amplitude was set at 0.6°.

The motion displacement curve of the stimulus was modulated by a sinusoidal sweep signal (chirp) or triangular sweep signal, and the frequency increased linearly to induce a continuous wide-band steady-state visual potential. Taking a sinusoidal motion stimulus as an example, the expression of the displacement curve was constructed as


(1) $$\text{y}\left(t\right)=\text{A c}os\left(2\pi \left(\frac{\text{a}}{2}\text{t}+\frac{{\text{f}}_{0}}{2}\right)\text{t}+\varphi_{0}\right)$$

where *A* is the motion amplitude, *a* is the frequency change rate, *φ_0_* is the initial phase, and *f*_0_ is the start motion reversal frequency. The motion reversal frequency, which indicates the frequency of motion direction conversion, is twice the frequency of a whole period of motion. The stimulation parameter setting of the SSMVEP paradigm is shown in Figure 1, the viewing angle of the motion stimulus was set at 5°, the motion amplitude was set at 0.6°, the initial phase was set at 0°, the duration of the stimulation trial was set at 8.5 s, the frequency change rate was set at about 2.47 Hz/s, the start motion reversal frequency was set at 7 Hz and the end frequency was set at 28 Hz corresponding to an average motion velocity of 8.4 deg/s (2*0.6°*7) to 33.6 deg/s (2*0.6°*28).

The motion reversal process is an important inducing factor for SSMVEPs, and the frequency of the SSMVEP generally takes the motion reversal frequency as the fundamental frequency. Therefore, the stimulus frequency mentioned in this study is equal to the motion reversal frequency.

**FIGURE 2:**
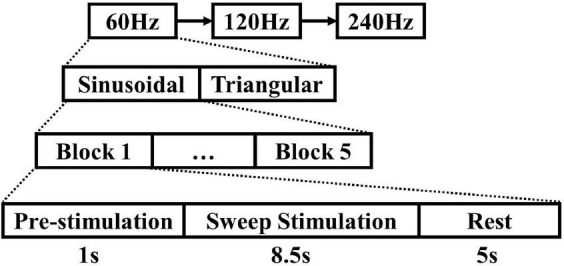
Experiment process. The duration of stimulation was 8.5 s, the rest interval was 5 s, and the experiment block with the same parameters was repeated 5 times.

To stabilize the visual evoked potential in advance, all participants watched the motion stimulus with the start frequency for 1 s before the formal experiment began. The experiment process is shown in Figure 2. In order to ensure the stability of the stimulation frequency, a photoelectric trigger device was used to test the visual paradigm before the formal experiment. The test results showed that only a few display frames have time deviations, and the error does not exceed 10-ms. When the formal experiment began, the stimulation frequency began to change. The duration of stimulation was 8.5 s, and the rest interval was 5 s. The experiment block with the same parameters was repeated 5 times. The motion paradigm was developed using MATLAB (MathWorks, Natick, United States) and Psychophysics Toolbox Version 3.

### Signal Analysis

#### Preprocessing of Electroencephalogram Data

A bandpass filter of 2–100 Hz and a 48–52 Hz notch filter were utilized to eliminate high-frequency interference, low-frequency drifts and power frequency interference of EEG signals. The five blocks were averaged to an 8.5-s data epoch for the next step in signal processing.

#### Canonical Correlation Analysis

Although Fourier transform is widely used for frequency detection with single-channel EEGs, it might still be sensitive to noise if the signal to be analyzed is from a single channel. Canonical correlation analysis (CCA) is an array signal processing method that can be used to calculate the underlying correlation between two sets of variables, it finds a pair of linear transforms for two sets and then maximizes their correlation.

CCA has been widely applied for frequency detection in multichannel visual-based BCIs (Lin et al., 2007; Zhang et al., 2020) due to its high efficiency, high robustness, high signal-to-noise ratio, and simple implementation (Bin et al., 2009; Kalunga et al., 2013; Nakanishi et al., 2015). Therefore, CCA was implemented to detect frequency components in our research.

Suppose that there are N frequencies *f_1_,f_2_,…,f_*N*_* that we need to analyze. To detect the stimulation frequency, two sets of signals are introduced into CCA. One set comprises the EEG signals *X* from several different recording channels. The other set comprises frequency signals *Y*_*i*_ (i = 1, …, N), denotes the reference signal and is constructed as


(2) $${\text{Y}}_{\text{i}}=\left(\frac{\sin\left(2\pi {\text{f}}_{\text{i}}n\right)}{\cos\left(2\pi {\text{f}}_{\text{i}}n\right)}\right),t=\frac{1}{\text{Fs}},\frac{2}{\text{Fs}},\cdots ,\frac{\text{K}}{\text{Fs}}$$

where *F*_*s*_ is the sampling rate and *K* is the number of sampling points. In this study, only the corresponding responses under different visual stimulations needed to be analyzed; therefore, the reference signals *Y*_*i*_ were only composed of sinusoid and cosinusoid pairs at the same frequency of the stimulus.

CCA can be used to find a pair of weight vectors *W*_*x*_ and *W*_*yi*_ to maximize the canonical correlation coefficient between linear transformations *X = X*^T^*W_*x*_* and *Y_*i*_ = Y_*i*_*^T^*W_*yi*_* by the following optimization problem:


(3) $$\underset{w_{x},w_{yi}}{\text{M}ax}\rho \left(x,{\text{y}}_{i}\right)=\frac{\text{E}\left[{\text{w}}_{x}^{T}{\text{XY}}_{i}^{T}{\text{w}}_{yi}\right]}{\sqrt{\text{E}\left[{\text{w}}_{x}^{T}{\text{XX}}^{T}{\text{w}}_{x}\right]\text{E}\left[{\text{w}}_{yi}^{T}{\text{Y}}_{i}{\text{Y}}_{i}^{T}{\text{w}}_{yi}\right]}}$$

where *E* represents the calculation of the expected value, ρ is the canonical correlation coefficient, and *x* and *y*_*i*_ are the first pair of canonical variables. *ρ(x,y_*i*_)* corresponds to the maximum canonical correlation coefficient between *x* and *y*_*i*_. When each canonical correlation coefficient of fi (i = 1, …, N) is calculated separately, the CCA response coefficient spectrum can be drawn by the maximum ρ of *N* canonical correlations.

This study used sliding window CCA spectrum analysis for time-frequency analysis. First, the 8.5-s EEG data in each block were superimposed in the time domain. Then, the EEG data were segmented according to a 0.75-s time window and a 0.25-s overlap length. A total of 32 segments were generated in this case, the frequency change range of each segment is about 0.66 Hz. Finally, CCA calculation was performed on the segmented data to obtain the correlation coefficient value. The response frequency corresponding to each segmented data was the average scanning stimulation frequency of the time window. The frequency range of the CCA coefficient spectrum analysis was set from 5 to 40 Hz, and the frequency interval was 0.2.

### Statistical Analysis

Two-way repeated measures analysis of variance (ANOVA) and one-way repeated measures ANOVA were used in this study to analyze the difference and agreement between different refresh rates and stimulation frequencies. *Post hoc* comparisons with the Bonferroni correction method for multiple comparisons were also used when necessary.

Before two-way or one-way repeated measures ANOVA was performed, outliers were removed by the studentized residual analysis, and the Shapiro-Wilk test was used to test whether each group of data obeyed a normal distribution. Mauchly’s test of sphericity was performed before repeated measures ANOVA was conducted. If Mauchly’s test of sphericity was violated, the data were corrected by the Greenhouse-Geisser estimates of sphericity. Two-way and one-way repeated measures ANOVA were carried out by SPSS (Version 22.0 IBM, Armonk, United States).

## Results

### Visual Evoked Potential Average Response Analysis

This subsection qualitatively analyzed the effect of refresh rate on the intensity of evoked response. First, the CCA coefficient spectrum analysis was preformed, which could present the response distribution of each subject under different stimulus conditions. Then the appropriate response frequency was selected to perform frequency response analysis, and the average evoked response intensity trend of all subjects was obtained. Finally, by dividing common EEG rhythms, the effect of refresh rate on the evoked response intensity under different frequency stimuli was presented.

#### Canonical Correlation Analysis Coefficient Spectrum Analysis of the Average Stimulus Response

**FIGURE 3:**
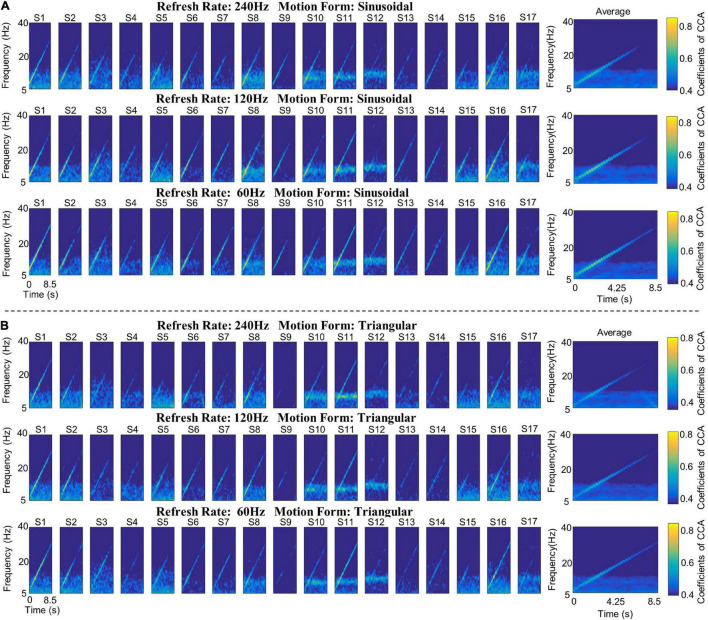
The CCA coefficient spectrum of the Sweep-SSMVEP paradigm. **(A)** The sinusoidal motion stimulation response. **(B)** The triangular motion stimulation response. Each row represents the stimulus response at the same refresh rate, each column (column 1–17) represents the stimulus response of the same participant, and the last column represents the average response of all participants under different refresh rates. In the CCA coefficient spectrum, the vertical axis indicates the response frequency, the horizontal axis indicates the stimulation duration, and the color indicates the value of the CCA coefficients.

The CCA coefficient spectrum analysis of the average stimulus response of all participants is presented in Figure 3. Figures 3A,B shows the sinusoidal motion and the triangular motion stimulation response, respectively.

The results of spectrum analysis demonstrate that the Sweep-SSMVEP paradigm evoked “single fundamental peak” responses. In other words, the fundamental frequency components of the Sweep-SSMVEP response (7–28 Hz) were prominent, whereas the higher-order harmonics were barely invisible. In addition, different motion forms and different refresh rates had little effect on high-order harmonic harmonics; therefore, in the subsequent analysis, the fundamental frequency response components were mainly considered evaluation indices.

#### Frequency Response Analysis of Fundamental Frequency

**FIGURE 4:**
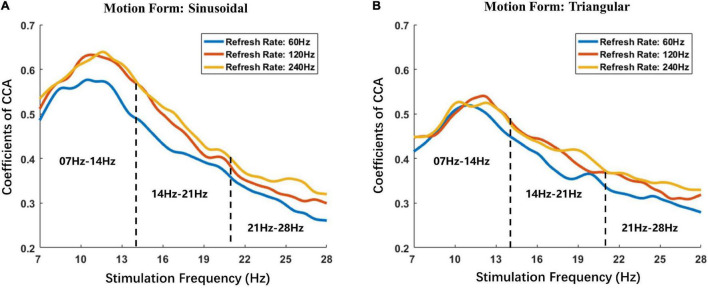
Fundamental average frequency responses of all participants. **(A)** The frequency response of sinusoidal motion stimulation. **(B)** The frequency response of triangular motion stimulation. The horizontal axis indicates the stimulation frequency at the corresponding time, and the vertical axis indicates the CCA coefficient under the corresponding stimulation frequency. The blue solid lines depict the average visual response of all participants to stimulation with a refresh rate of 60 Hz, the red solid line depicts the average visual response to stimulation with a refresh rate of 120 Hz and the yellow solid line depicts the average visual response to stimulation with a refresh rate of 240 Hz. The black dotted line depicts the frequency divisions.

The average fundamental frequency responses of all participants are presented in Figure 4. Figures 4A,B show the frequency response of sinusoidal motion stimulation and triangular motion stimulation, respectively. To compare the effect of the refresh rate of the monitor on the evoked potential response under different frequencies, the stimulation frequencies were divided into three ranges according to the EEG rhythm: alpha wave (7–14 Hz), low beta wave (14–21 Hz) and middle beta wave (21–28 Hz).

The changing trend of the fundamental frequency response of the Sweep-SSMVEP paradigm was similar to that of the flicker SSVEP paradigm. The response amplitude reached the highest value when the stimulation frequency was approximately 10 Hz and then dropped as the stimulation frequency increased. Furthermore, the results of frequency responses demonstrated that refresh rates of visual motion stimulation significantly influence the intensity of the evoked potential and that the law of effect is also related to the frequency or form of stimulation. The results show that the sinusoidal motion stimulation response intensities under refresh rates of 120 Hz (Average CCA: 0. 0.4623) and 240 Hz (Average CCA: 0. 0.4771) were both higher than that under a refresh rate of 60 Hz (Average CCA: 0.4226) with an average increase of 8.8 and 12.4%, respectively. Moreover, the triangular motion stimulation response intensity under refresh rates of 120 Hz (Average CCA: 0.4162) and 240 Hz (Average CCA: 0.4236) were also both higher than that under a refresh rate of 60 Hz (Average CCA: 0.3915), with an average increase of 6.5 and 8.8%, respectively.

#### Average Response Intensity in Different Stimulation Frequency Bands

**FIGURE 5:**
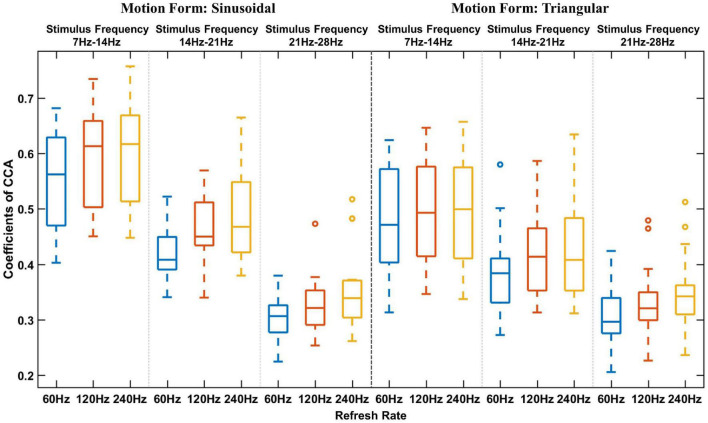
Average stimulation response boxplot of different frequencies. The horizontal axis indicates the refresh rate, and the vertical axis indicates the average CCA coefficient. The black dotted line is used to separate different motion forms, and the blue dotted line is used to separate different stimulation frequencies.

As shown in the average stimulation response boxplot (Figure 5), the alpha wave (7–14 Hz), low beta wave (14–21 Hz), and middle beta wave (21–28 Hz) responses of all participants were averaged. To distinguish parameters, a different color was used to indicate different refresh rates. The box plot results suggest that, in general, a high refresh rate can induce a higher visual potential response than a low refresh rate, and sinusoidal motion stimulation can induce a higher visual potential response than triangular motion stimulation. These data were used in subsequent statistical analyses.

### The Effect of the Refresh Rate of the Monitor on the Sinusoidal Visual Motion Stimulation Response

The two-way repeated measures ANOVA was applied in this subsection to analyze the effect of the refresh rate on the sinusoidal visual motion stimulation response. First, it was necessary to determine the interaction effect of the refresh rate and stimulation frequency, that is, to find out whether the refresh rate have a differentiated effect under different frequency stimulations. When the interaction effect between refresh rate and stimulation frequency was determined, one-way repeated measures ANOVA was used to perform simple effect analysis in each frequency band, respectively, which could determine the response intensity significant difference under different refresh rates. If the one-way repeated measures ANOVA show that the refresh rate will have significant different effects on the evoked response in a certain stimulation frequency range, then the *post hoc* comparisons analysis could be further carried out to determine the refresh rate response intensity difference between each other, finally obtained specific statistical analysis results.

#### Analysis of the Interaction Effect of the Refresh Rate and Stimulation Frequency

The CCA coefficient data of the sinusoidal stimulation response satisfied the conditions of two-way repeated measures ANOVA, and the distribution of response data obeyed a normal distribution and satisfied the sphericity property [Mauchly’s test of sphericity, χ^2^(9) = 4.17, *P* = 0.043 > 0.05].

The outcomes of the analysis suggest that the interaction effect of the refresh rate and stimulation frequency had a statistically significant effect on the evoked response to sinusoidal motion stimulation [*F*(4, 56) = 3.30, *P* = 0.017 < 0.05, η*_*p*_*^2^= 0.19]. Therefore, it was possible to analyze evoked response changes with different refresh rates under three frequency band sinusoidal motion stimulations separately.

#### The Simple Effect Analysis of Refresh Rate in Each Frequency Band

One-way repeated measures ANOVA was used to analyze the simple effect of refresh rate in each frequency band. Mauchly’s test of sphericity was also used to evaluate whether the sphericity assumption was violated. The results showed that the CCA coefficient data of 7–14 Hz sinusoidal stimulation responses [χ^2^(2) = 6.83, *P* = 0.033 < 0.05] and 21–28 Hz stimulation responses [χ^2^(2) = 6.16, *P* = 0.046 < 0.05] violated Mauchly’s test of sphericity, and the CCA coefficient data of 14–21 Hz stimulation responses [χ^2^(2) = 2.41, *P* = 0.300 > 0.05] were not violated. Then, the Greenhouse-Geisser estimates of sphericity were used to correct the CCA coefficient data (ε_7–14 *Hz*_ = 0.87, ε_21–28 *Hz*_ = 0.73).

The outcomes of one-way repeated measures ANOVA suggested that the refresh rate had a statistically significant simple effect on the evoked response under sinusoidal stimulation in the 7–14 Hz frequency band [*F*(1.46, 23.43) = 17.24, *P* < 0.001, η*_*p*_*^2^= 0.52], 14–21 Hz frequency band [*F*(2, 32) = 15.16, *P* < 0.001, η*_*p*_*^2^= 0.49] and 21–28 Hz frequency band [*F*(1.452, 20.33) = 12.188, *P* = 0.01, η*_*p*_*^2^ = 0.47].

#### *Post hoc* Comparisons of the Refresh Rate in Each Frequency Band

The differences in evoked responses under different refresh rates in each stimulation frequency band were compared by *post hoc* comparisons. The results are shown in Table 1. The column of differences of evoked responses indicates the difference between different refresh rates. The asterisk in the column of significance indicates that the difference is statistically significant at the level of α = 0.05.

**TABLE 1:** The *post hoc* comparison results of sinusoidal motion stimulation with different refresh rates.

Stimulation frequency	Refresh rate	Average CCA coefficient mean (SD)	Differences of evoked responses		Significance
			Mean (S.E.)	95% CI	
**7–14 Hz**	60 Hz	0.546 (0.092)	120–60 Hz: 0.047 (0.009)	[0.023 0.072]	P < 0.001*
	120 Hz	0.594 (0.094)	240–60 Hz: 0.052 (0.012)	[0.019 0.085]	P = 0.002*
	240 Hz	0.599 (0.100)	240–120 Hz: 0.005 (0.007)	[−0.014 0.024]	*P = 1*

**14–21 Hz**	60 Hz	0.418 (0.053)	120–60 Hz: 0.047 (0.009)	[0.022 0.072]	P < 0.001*
	120 Hz	0.465 (0.059)	240–60 Hz: 0.062 (0.013)	[0.026 0.097]	P = 0.001*
	240 Hz	0.480 (0.079)	240–120 Hz: 0.015 (0.012)	[−0.018 0.047]	*P = 0.73*

**21–28 Hz**	60 Hz	0.295 (0.032)	120–60 Hz: 0.019 (0.005)	[0.005 0.033]	P = 0.006*
	120 Hz	0.314 (0.035)	240–60 Hz: 0.038 (0.009)	[0.012 0.063]	P = 0.004*
	240 Hz	0.332 (0.054)	240–120 Hz: 0.018 (0.008)	[−0.03 0.039]	*P = 0.10*

*p < 0.05.

**FIGURE 6:**
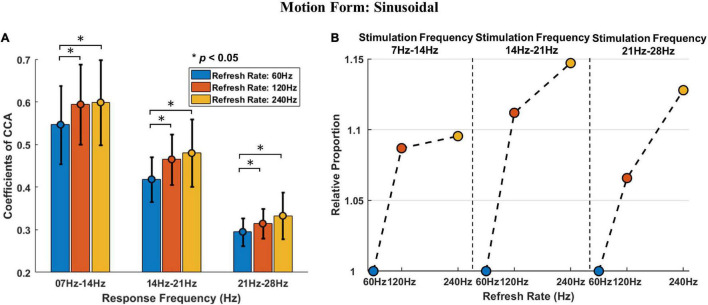
The *post hoc* comparison results and relative proportion comparison of sinusoidal motion stimulation. **(A)** The histogram of sinusoidal motion stimulation-evoked responses, including the mean and standard deviation. The asterisks indicate that the difference is statistically significant at the level of α = 0.05. **(B)** The relative proportion of sinusoidal stimulation-evoked responses under different refresh rates in each stimulation frequency range. The vertical axis indicates the relative proportion, the horizontal axis indicates the refresh rate, and the black dotted thin line is used to separate different stimulation frequencies.

Figure 6 is a graphical display of the data in Table 1. Figure 6A shows the histogram of sinusoidal motion stimulation-evoked responses, including the mean and standard deviation. Different colors are used to indicate different refresh rates. Figure 6B shows the relative proportion of evoked responses under different refresh rates in each stimulation frequency. The relative average CCA coefficient of evoked responses under a refresh rate of 60 Hz was set at 1, which allowed the calculation and application of the relative average CCA coefficient under 120 and 240 Hz.

The results of *post hoc* comparisons demonstrate that the sinusoidal motion stimulation response intensities under refresh rates of 120 and 240 Hz were both higher than that under a refresh rate of 60 Hz with an average increase of 8.8 and 12.4%, respectively, and the differences were statistically significant. The response intensity was also higher under a refresh rate of 240 Hz than that under 120 Hz refresh rate, with an average increase of 3.3%, but the difference was not statistically significant.

Furthermore, the stimulation frequency and refresh rate had a significant interactive effect on the visual evoked potential response. As shown in Figure 6B, the response intensity differences between the 60 Hz refresh rate and 120 Hz refresh rate under all frequencies of stimulation were remarkable. However, the response intensity difference between the 120 Hz refresh rate and the 240 Hz refresh rate varied drastically with stimulation frequency. That is, the response intensity difference was minor at lower frequency stimulation; as stimulation frequency increased, the difference became larger, and the overall effect trend of the refresh rate on response intensity became linear.

### The Effect of the Refresh Rate on the Triangular Visual Motion Stimulation Response

The analysis of the effect of the refresh rate on the triangular visual motion stimulation response was similar to the analysis process of sinusoidal motion stimulation, the methods are as follows: interaction effect analysis, simple effect analysis and *post hoc* comparisons.

#### Analysis of the Interaction Effect of the Refresh Rate and Stimulation Frequency

For triangular motion stimulation, the data satisfied the sphericity property [Mauchly’s test of sphericity, χ^2^(9) = 8.21, *P* = 0.52 > 0.05], which mean the data of triangular wave stimulation responses met the conditions of two-way repeated measurement ANOVA.

The outcomes of two-way repeated measures ANOVA also demonstrated that the interaction effect of the refresh rate and stimulation frequency was statistically significant [*F*(4, 56) = 2.532, *P* = 0.05, η*_*p*_*^2^ = 0.15].

#### Simple Effect Analysis of the Refresh Rate in Each Frequency Band

The results showed that the CCA coefficient of triangular stimulation response data at frequencies of 7–14 Hz [χ^2^(2) = 0.55, *P* = 0.76 > 0.05], 14–21 Hz [χ^2^(2) = 0.19, *P* = 0.91 > 0.05] and 14–21 Hz [χ^2^(2) = 4.88, *P* = 0.087 > 0.05] did not violate Mauchly’s test of sphericity.

The outcomes of one-way repeated measures ANOVA demonstrated that the refresh rate had a statistically significant simple effect on the evoked response at the 14–21 Hz frequency band [*F*(2, 30) = 10.63,*P* < 0.001, η*_*p*_*^2^= 0.415] and 21–28 Hz frequency band [*F*(2, 28) = 13.07, *P* < 0.001, η*_*p*_*^2^= 0.483], and the simple effect of the refresh rate on the 7–14 Hz evoked response was not statistically significant [*F*(2, 32) = 2.456, *P* = 0.1 > 0.05, η*_*p*_*^2^ = 0.13].

#### *Post hoc* Comparisons of the Refresh Rate in Each Frequency Band

Consequently, only *post hoc* comparisons of responses to 14–21 Hz and 21–28 Hz stimulations were performed. The analysis results of triangular motion stimulation with different refresh rates are shown in Table 2. The asterisk in the column of significance indicates that the difference is statistically significant at the level of α = 0.05.

**TABLE 2:** The *post hoc* comparison results of sinusoidal motion stimulation with different refresh rates.

Stimulation frequency	Refresh rate	Average CCA coefficient mean (SD)	Differences of evoked responses		Significance
			Mean (S.E.)	95% CI	
**7–14 Hz**	60 Hz	0.374 (0.060)	120–60 Hz: 0.034	−	−
	120 Hz	0.407 (0.065)	240–60 Hz: 0.038	−	−
	240 Hz	0.412 (0.071)	240–120 Hz: 0.004	−	−

**14–21 Hz**	60 Hz	0.374 (0.060)	120–60 Hz: 0.034	[0.009 0.058]	P = 0.006*
	120 Hz	0.407 (0.065)	240–60 Hz: 0.038	[0.015 0.064]	P = 0.001*
	240 Hz	0.412 (0.071)	240–120 Hz: 0.004	[−0.021 0.03]	*P = 1*

**21–28 Hz**	60 Hz	0.295 (0.038)	120–60 Hz: 0.022	[0.008 0.035]	P = 0.002*
	120 Hz	0.316 (0.038)	240–60 Hz: 0.037	[0.014 0.061]	P = 0.002*
	240 Hz	0.332 (0.050)	240–120 Hz: 0.016	[−0.005 0.037]	*P = 0.18*

*p < 0.05.

**FIGURE 7:**
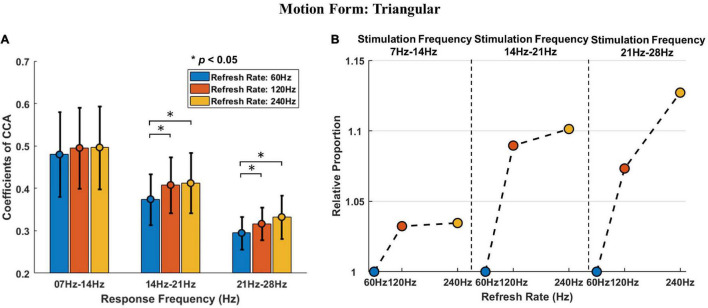
The *post hoc* comparison results and relative proportion comparison of triangular motion stimulation. **(A)** The histogram of triangular motion stimulation-evoked responses, including the mean and standard deviation. The asterisks indicate that the difference is statistically significant at the level of α = 0.05. **(B)** The relative proportion of triangular stimulation-evoked responses under different refresh rates in each stimulation frequency range. The vertical axis indicates the relative proportion, the horizontal axis indicates the refresh rate, and the black dotted thin line is used to separate different stimulation frequencies.

Figure 7 is a graphical display of the data in Table 2. Figure 7A shows the histogram of triangular motion stimulation-evoked responses, including the mean and standard deviation. Figure 7B shows the relative proportion of evoked responses under different refresh rates in each stimulation frequency range.

Similar to the effect of refresh rate under sinusoidal motion stimulation, the results demonstrate that the triangular motion stimulation response intensity under refresh rates of 120 and 240 Hz were also both higher than that under a refresh rate of 60 Hz, with an average increase of 6.5 and 8.8%, respectively. However, this statistical significance only occurred in the triangular motion stimulation with middle (14–21 Hz) and high (21–28 Hz) frequencies. The response intensity under the 240 Hz refresh rate response intensity was also higher than that under the refresh rate of 120 Hz, with an average increase of 2.1%, and the difference was not statistically significant.

The interactive effect of stimulation frequency and refresh rate under triangular motion stimulation was also similar to the interactive effect under sinusoidal motion stimulation. The response intensity differences between the 60 Hz refresh rate and the 120 Hz refresh rate were notable, whereas the response intensity difference between the 120 Hz refresh rate and the 240 Hz refresh rate was minor under lower frequency stimulation. As the stimulation frequency increased, the difference became larger. This conclusion means that the increase in refresh rate can improve the response intensity of motion stimulation and enhance the perceptual ability of visual motion. This conclusion further confirms that the refresh rate enhances motion perception.

These conclusions from the analysis of sinusoidal and triangular motion stimulation responses mean that an increased refresh rate can improve the response intensity of motion stimulation and enhance the perceptual ability of visual motion.

### The Effect of the Motion Form in Each Refresh Rate Group

The analysis of the effect of the motion form was similar to the analysis process of the effect of the refresh rate, the methods are as follows: interaction effect analysis and simple effect analysis. Besides, it is worth pointing out that the *post hoc* comparison was not applicable, because there were only two motion parameters in simple effect analysis.

#### Analysis of the Interaction Effect of the Motion Form and Stimulation Frequency

Similar to the above analysis, the data or corrected data of different motion forms in each refresh rate group were tested to meet the conditions of two-way and one-way repeated measurement ANOVA.

The result of Mauchly’s test of sphericity showed that the CCA coefficient data of the refresh rate of 60 Hz [χ^2^(2) = 7.84, *P* = 0.02 < 0.05] did not violate Mauchly’s test of sphericity, and the data of the refresh rates of 14–21 Hz [χ^2^(2) = 2.59, *P* = 0.27 > 0.05] and 14–21 Hz [χ^2^(2) = 2.02, *P* = 0.364 > 0.05] violated Mauchly’s test of sphericity. After data correction (ε_60*Hz*_ = 0.87), two-way repeated measures ANOVA was performed on the data.

The outcomes of two-way repeated measures ANOVA suggest that the interaction effect of motion form and stimulation frequency under each refresh rate was statistically significant [*F*_60 *Hz*_ (1.42, 17.11) = 3.994, *P*_60 *Hz*_ = 0.048 < 0.05, η*_*p*_*^2^ = 0.25; *F*_120 *Hz*_ (2, 24) = 6.69, *P*_120 *Hz*_ = 0.005 < 0.05, η*_*p*_^2^* = 0.358; *F*_240 *Hz*_ (2, 24) = 5.78, *P*_240 *Hz*_ = 0.009 < 0.05, η*_*p*_*^2^ = 0.325].

The outcomes of the simple effect analysis of motion forms in each stimulation frequency band are shown in Table 3. The column of differences of evoked responses indicates the difference between sinusoidal stimulation and triangular stimulation. The asterisk in the column of significance in Table 3 indicates that the difference is statistically significant at the level of α = 0.05.

**TABLE 3:** The simple effect analysis results of motion forms.

Refresh rate	Stimulation frequency	Motion form	Average CCA coefficient mean (SD)	Differences of evoked responses (sinusoidal-triangular)		Significance
				Mean	95% CI	
**60 Hz**	7–14 Hz	Sinusoidal	0.54 (0.091)	0.067	[−0.009 0.14]	*P = 0.079*
		Triangular	0.46 (0.1)			
	14–21 Hz	Sinusoidal	0.42 (0.05)	0.049	[0.008 0.089]	P = 0.022*
		Triangular	0.36 (0.06)			
	21–28 Hz	Sinusoidal	0.3 (0.028)	0.007	[−0.027 0.041]	*P = 0.65*
		Triangular	0.29 (0.039)			

**120 Hz**	7–14 Hz	Sinusoidal	0.58 (0.094)	0.099	[0.03 0.17]	P = 0.008*
		Triangular	0.47 (0.095)			
	14–21 Hz	Sinusoidal	0.46 (0.061)	0.059	[0.008 0.11]	P = 0.026*
		Triangular	0.4 (0.065)			
	21–28 Hz	Sinusoidal	0.32 (0.031)	0.003	[−0.032 0.037]	*P = 0.87*
		Triangular	0.31 (0.037)			

**240 Hz**	7–14 Hz	Sinusoidal	0.58 (0.099)	0.102	[0.027 0.18]	P = 0.01*
		Triangular	0.47 (0.098)			
	14–21 Hz	Sinusoidal	0.47 (0.08)	0.072	[0.008 0.14]	P = 0.029*
		Triangular	0.4 (0.071)			
	21–28 Hz	Sinusoidal	0.34 (0.055)	0.013	[−0.037 0.063]	*P = 0.58*
		Triangular	0.32 (0.036)			

*p < 0.05.

**FIGURE 8:**
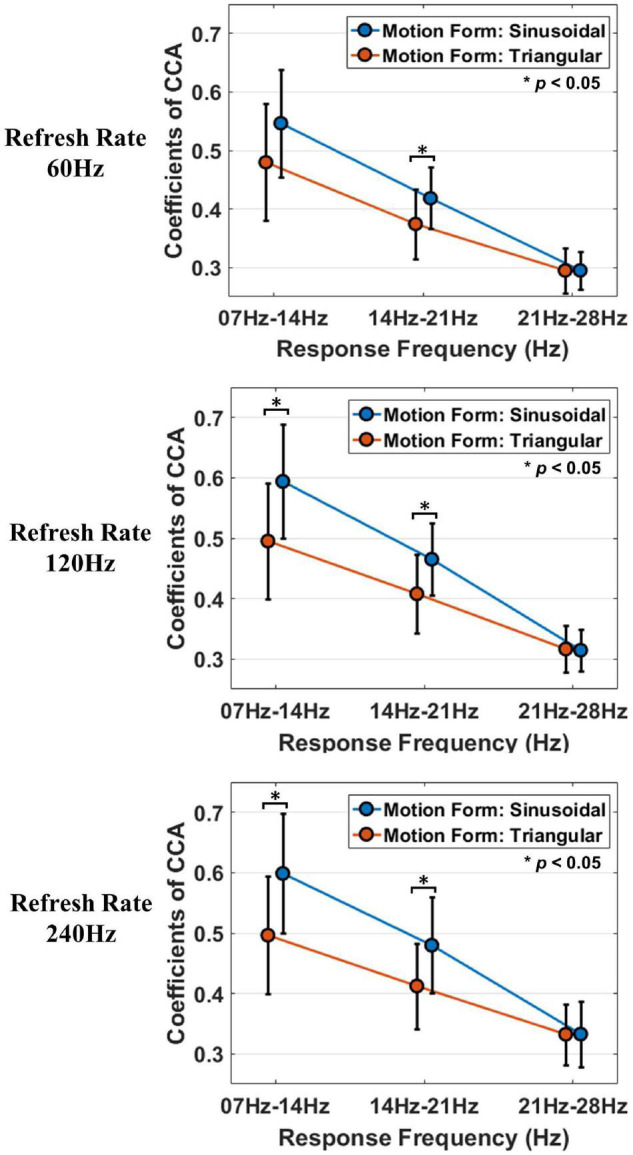
The simple effect analysis results of motion forms in each refresh rate group. Different colors are used to indicate different motion forms, and the asterisks indicate that the difference is statistically significant at the level of α = 0.05. The vertical axis indicates the coefficients of CCA, and the horizontal axis indicates the stimulation frequency.

Figure 8 is a graphical display of the data in Table 3. Figure 8 shows the mean and standard deviation of stimulation-evoked responses in each refresh rate group.

The outcomes of simple effect analysis of motion forms suggest that the simple effects of motion forms at 14–21 Hz were statistically significant under a 60 Hz refresh rate, the simple effects of motion forma at 7–4 Hz and 14–21 Hz were statistically significant under a 120 Hz refresh rate, the simple effects of motion forms at 7–14 Hz and 14–21 Hz were statistically significant in the 240 Hz refresh rate group.

However, it is worth pointing out that although the simple effect of motion forms at the frequency of 7–14 Hz and the refresh rate of 60 Hz was not statistically significant (*P* = 0.079 > 0.05), the difference between the sinusoidal stimulation response and triangular stimulation response was noteworthy. The reason for this outcome may be the volatility of variance due to the sample size.

These results demonstrate that the visual evoked response intensity of sinusoidal motion stimulation is significantly different from that of triangular motion stimulation. However, the difference is also related to the stimulation frequency; that is, the response difference is significant at a frequency in low and middle ranges (7–14 Hz and 14–21 Hz) whereas it is not significant at a high-range frequency (21–28 Hz).

#### Difference of Various Motion Forms

**FIGURE 9:**
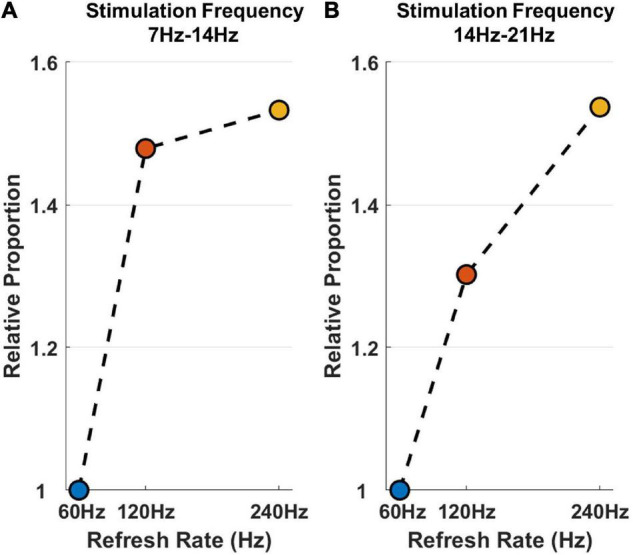
The relative proportion of the response intensity difference between different motion forms. **(A)** The difference at the frequency of 7–14 Hz. **(B)** The difference at the frequency of 14–21 Hz. The horizontal axis and different colors indicate stimulation frequencies, and the vertical axis indicates the relative proportion of the response intensity difference between different motion forms.

The relative proportion of the response intensity difference between different motion forms is shown in Figure 9. The response intensity difference was calculated by subtracting the average response of the triangular stimulation from the average response of sinusoidal motion stimulation, then setting the relative difference of the 60 Hz rate refresh to 1, and calculated and achieved relative proportion of other relative difference under 120 and 240 Hz.

Figure 9A shows the difference at frequencies of 7–14 Hz, and Figure 9B shows the difference at frequencies of 14–21 Hz. Since the difference in the response intensity of different motion form stimulations at a high frequency was not significant, the relative difference at frequencies of 21–28 Hz is not presented in Figure 9.

At frequencies of 7–14 Hz, the response difference under refresh rates of 240 and 120 Hz between sinusoidal motion and triangular motion rises by 48 and 53% on average, respectively, compared with that under a refresh rate of 60 Hz. At frequencies of 14–21 Hz, the response difference rises by 30 and 54% on average, respectively. The results demonstrate that increasing the refresh rate can increase the difference in motion visual evoked potential between sinusoidal stimulation and triangular stimulation; in particular, this effect is prominent when the motion stimulation frequency is not high. This conclusion further confirms that the refresh rate enhances motion perception. However, since data were limited by the maximum refresh rate in this study, the difference in high-frequency stimulation between different motion forms was insignificant, and it cannot be indicated that increasing the refresh rate can effectively improve the perception and ability to distinguish of high-frequency motion.

## Discussion

### Selection of Monitor Parameters

CRT monitors have the characteristics of low latency and high stability, and they have long been the standard equipment used in visual perception research (Wiens and Öhman, 2007). However, LCD monitors have gradually become mainstream equipment with the improvement of production technology; they are more energy-efficient and compact and show little or no visual flicker. Many studies have proven that the performance of LCD monitors is also close to that of CRT displays (Kihara et al., 2010; Lagroix et al., 2012; Bognár et al., 2016; Zhang et al., 2018; Rohr and Wagner, 2020). Therefore, the LCD monitor was chosen as the experimental equipment in this study.

A low delay response time and high refresh rate are both effective measures to improve the performance of motion stimulation (Claypool and Claypool, 2007, 2009; Spjut et al., 2019; Denes et al., 2020). The effect of the refresh rate on visual perception was a major concern in this study. To avoid interference from latency factors, a low-latency LCD monitor (ASUS ROG PG258Q) with multiple optional refresh rates was chosen as the experimental equipment. When the overdrive setting parameter of the monitor was set to “normal,” the average gray-to-gray (GtG) delay response time of this monitor was approximately 4.9, 3.3, and 2.9 ms when the refresh rate reached 60, 120, and 240 Hz, respectively.^1^ The GtG delay response times were all less than the refresh time, and the ghosting artifacts caused by the latency of response time were slight, so the negative impact of the delay response time was not considered.

The ultralow motion blur (ULMB) function of the monitor was not enabled in the experiment. Although this function helps reduce motion blur to a degree (Zhang et al., 2018), it is a technology only available in high-end monitoring and causes flicker sensation and visual fatigue. In the experimental SSMVEP paradigm, the stimulus was internal texture motion in a circle with fixed size and position, the participants were required to gaze at the target stimulus without eye movement, and the positive impact of the ULMB technique was further limited.

### The Effect of the Refresh Rate on the Intensity of Steady-State Response, Which Can Be Called Steady-State Motion Visual Evoked Potentials

SSMVEPs are induced by the perception of stable frequency visual motion stimulation. In previous studies, it was found that the SSMVEP has the characteristic of a single peak, the evoked response energy is concentrated (Han et al., 2018). This characteristic makes the steady-state motion paradigm very suitable as a “probe” for non-invasive visual perception research. At present, a vision assessment method using steady-state motion visual evoked potential has been proposed (Zheng et al., 2019) to achieve an objective and quantitative assessment of visual acuity. The experimental results show that the correlation and agreement between objective SSMVEP and subjective FrACT (Freiburg Visual Acuity and Contrast Test) acuity were all good (Zheng et al., 2020a), demonstrating good performance in visual perception detection for the motion visual stimulation paradigm.

Due to the refresh interval between display frames, the lower the rendering refresh rate is, the greater the possibility of causing motion blur and dispersion, which will have a negative impact on the elicitation of visual potentials. Therefore, the change in the response intensity of visual evoked potentials can be used to measure whether the display system can correctly present the motion stimulus. However, it is important to note that the amplitude of EEG-based SSVEPs or SSMVEP is very unreliable at very high frequencies (>40 Hz) or very low frequencies (<2 Hz), and the assessment method in this study cannot be used in this case.

The results of the study demonstrate that the refresh rate has a significant positive effect on the perception response of visual motion stimulation at different frequencies. The intensity of the visual evoked potential under high refresh rate stimulation is always higher than the intensity of that under low refresh rate stimulation. Similar to the results of previous research literature (DoVale, 2017), there is a range in which a plateau of slow growth is observed, the effect of refresh rate has obvious diminishing returns. The positive effect of refresh rate is most significant when the refresh rate is increased from 60 to 120 Hz, and then the positive effect gradually gets into the realms of diminishing returns as the refresh rate range continues to increase above 120 Hz. This trend has no concern with the form of motion stimulation.

In addition, the diminishing return is also related to the stimulation frequency, and the attenuation effect of improving the evoked response at low-frequency stimulation is more obvious than that at high-frequency stimulation. In other words, under high frequency (21–28 Hz) stimulation, the increment of response between 120 and 60 Hz refresh rates is similar to that between 240 and 120 Hz refresh rates. However, the increment of the response between the 120 and 60 Hz refresh rates was much larger than that between the 240 and 120 Hz refresh rates under low-frequency (7–14 Hz) stimulation.

The reason for this phenomenon may be related to the adequacy of the spatiotemporal sampling of the stimulus motion. Adelson and Bergen (1985) developed a model of motion detection in which spatiotemporal filtering is used to detect motion energy of luminance-defined motion. Fujii et al. (2018) reported smoother motion in high frame rate content should activate the central nerve of vision more effectively because it produces more motion energy than low frame rate stimulus based on these models. This mechanism explains why there is a significant interaction between the refresh rate and the stimulation frequency in our experiment. In other words, high-frequency motion under low refresh rate has poor smoothness, increasing the refresh rate in this case can obviously improve the motion energy of visual stimulation, but increasing the refresh rate is of little significance for smoother motion.

It has been long known that the mammalian visual system is highly sensitive to motion, even when presented briefly. The middle temporal visual area is a region of the extrastriate visual cortex in primates that has been demonstrated to be critical for motion vision. Area MT has among the shortest response latencies in the extrastriate cortex (Schmolesky et al., 1998; Born and Bradley, 2005). In 2011, researchers used three refresh rates to investigate how changes in the CRT (cathode ray tube) temporal stimulus affect cortical responses in tree shrew V1 (the primary visual cortex), they find that refresh rate had a large impact on firing rate and the amplitude of LFP (120 Hz > 90 Hz > 60 Hz). Since mean firing rate is positively correlated with refresh rate, V1 acts like a high-pass filter for sparse noise stimuli as a function of refresh rate (Potter et al., 2014). Furthermore, researchers found the minimum timescale for motion encoding by ganglion cells of cat retinal was 4.6 ms and depended non-linearly on temporal frequency in 2011 (Borghuis et al., 2019). These anatomical evidences from retinal nerves to higher visual cortex nerves demonstrated that the perception frequency of human vision for continuous motion may be much higher than previously speculated. Therefore, consider of the display hardware burden, we choose 120 Hz refresh rate as a conservative estimate of the optimal motion presentation parameters.

### The Effect of the Refresh Rate on the Ability to Distinguish Motion Forms

The visual evoked responses caused by various motion forms are different (Teng et al., 2011; Grgič et al., 2016; Labecki et al., 2016). In this study, the experiments verified that there were significant differences in the intensity of visual evoked potentials between sinusoidal and triangular motion stimulation, and the response of sinusoidal motion stimulation was higher than that of triangular motion stimulation in general. The reason may be the difference in continuity in the motion reversal process. The motion reversal process is an important way to induce SSMVEPs, and a continuous and clear motion reversal process can improve the evoked response. In the triangular motion stimulation, the absolute value of speed always remains constant, and the rendering points are evenly distributed in the motion trajectory. In the sinusoidal motion stimulation, the rendering points are more concentrated around the reversal position, and the motion reversal process is more continuous, so the inducing effect of sine motion stimulation is superior.

The results of this analysis show that the difference in evoked potential response intensity between different motion forms increases with the refresh rate. In other words, the increase in refresh rate can improve the ability to distinguish between similar visual motions. This conclusion further demonstrates the positive effect of the rate refresh on the perception response to visual motion stimulation. Moreover, the difference in response intensity between different motion stimulations is also affected by the stimulation frequency. The difference is significant under low-frequency stimulation, but as the frequency increases, the difference decreases until it is not significant. Therefore, the changes in response difference under medium- and low-frequency stimuli were mainly analyzed to evaluate the effect of the refresh rate.

**FIGURE 10:**
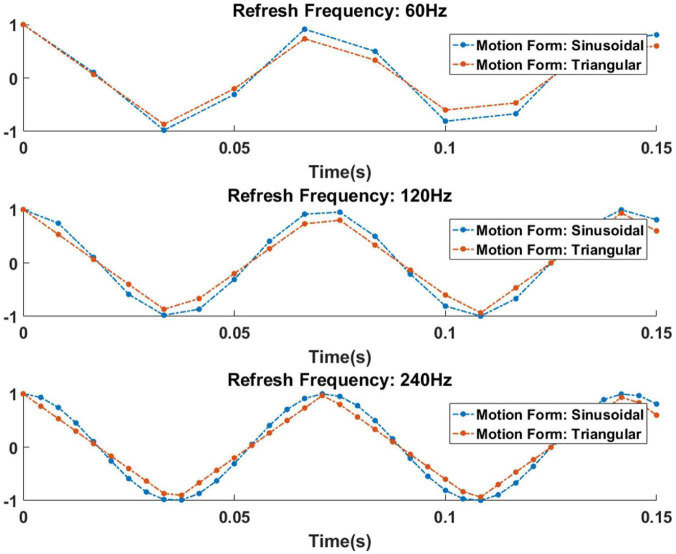
The similarity of sinusoidal and triangular motion at different refresh rates. The vertical axis indicates displacement of stimulation, and the horizontal axis indicates stimulation time.

The reason for this phenomenon is also obvious, as shown in Figure 10. In this figure, the results of a 14 Hz (motion reversal frequency) motion stimulation process of sinusoidal and triangular structures at different refresh rates are depicted. It is difficult to distinguish the displacement details of different forms under a low refresh rate. With the increase in the refresh rate, the details of displacement are gradually improved, and different motion forms can be distinguished.

### Refresh Rate Selection With Different Stimulation Frequencies

The above analysis determined the effect of the refresh rate on the response intensity of evoked potentials with different stimulation frequency bands. Within the frequency range (7–28 Hz) set by the experiment, the response intensity and motion discrimination ability at a refresh rate of 60 Hz are significantly lower than those at refresh rates of 120 and 240 Hz. This result suggests that a monitor with a refresh rate of 60 Hz has a limited ability to present fast motion stimulation. Therefore, unless special displays such as VR devices must be used or the frequency of motion stimulation is very low, the findings study signify that a monitor with a high refresh rate (120 Hz or above) should be chosen to ensure accurate motion presentation in visual perception or BCI experiments.

Furthermore, although the positive effect gradually enters the realm of diminishing returns as the refresh rate range continues to increase above 120 Hz, the decay trend is not significant when the stimulation frequency is high, and choosing a monitor with an ultrahigh refresh rate (240 Hz or above) is also of considerable significance. It is recommended, if conditions permit, to choose a monitor with an ultrahigh refresh rate according to the motion frequency or speed.

This study proposes a visual motion perception assessment method based on visual electrophysiological signals. A flicker-free Sweep-SSMVEP paradigm was designed and utilized to assess the effect of the refresh rate on motion stimulation of different frequencies. The results demonstrate that the refresh rate had a positive effect and improved visual motion perception, and the refresh rate also had a significant interaction between the refresh rate and stimulation frequency. In future studies, we will examine the impact of motion perception with eye movement on visual evoked potentials and improve the assessment method to make it suitable for visual motion perception at extreme frequencies or extreme velocities.

### Visual Fatigue and Limitation

In the research of visual perception, long-term viewing of strong stimuli may cause adaptation effect (Heinrich and Bach, 2001; Priebe et al., 2002) and visual fatigue (Cao et al., 2014), resulting in changes of the evoked potential amplitude that interfere with the ability to achieve an accurate motion perception response.

Therefore, we improved the stimulation pattern to minimize the limitation of visual fatigue. The steady-state motion reversal stimulation was used as stimulation pattern in the visual motion perception experiment, the steady-state motion reversal stimulation can overcome the high susceptibility to adaptation (Heinrich and Bach, 2003) and also has a good long-term fatigue resistance (Xie et al., 2016; Zheng et al., 2020b). Furthermore, the sweep signal was used to modulated the motion stimulation, which greatly reduced the experiment time. Therefore, the total visual stimulation time of each subject is less than 5 min, there would be no obvious fatigue problems during this period of time.

However, visual fatigue still limits the development of this research. The experiment only uses the common refresh rate of 60, 120, and 240 Hz. Although the results show that the refresh rate of 60–120 Hz has a significant effect on the motion visual response, in order to control the duration of experiment, accurate segmentation of the refresh rate is not performed which leads to an inability to determine the influence trend detail of refresh rate.

## Conclusion

The implications of this study are that it proposes an objective, reliable, visual electrophysiological method and assesses the effect of the refresh rate on motion stimulation at different frequencies with the method. The results demonstrated that an increase in the refresh rate significantly improved the intensity of sinusoidal motion visual evoked potentials at the three stimulation frequency ranges of 7–14 Hz [*F*(1.46, 23.43) = 17.24, *P* < 0.001, η^2^= 0.52], 14–21 Hz [*F*(2, 32) = 15.16, *P* < 0.001, η^2^= 0.49], and 21–28 Hz [*F*(1.452, 20.33) = 12.188, *P* = 0.01, η^2^= 0.47]. The intensity of the response at refresh rates of 240 and 120 Hz increased by 8.8 and 12.4% on average, respectively, compared with that at a refresh rate of 60 Hz. There was a significant interaction between the refresh rate and sinusoidal motion frequency [*F*(4, 56) = 3.30, *P* = 0.017 < 0.05, η^2^= 0.19], and the effect of the refresh rate more easily reached diminishing returns at lower frequencies. Furthermore, the increased refresh rate also had the potential to enhance the ability to perceive similar motion. Therefore, a refresh rate of at least 120 Hz is recommended for motion visual perception experiments to ensure a better stimulation effect, if the motion frequency or velocity is high, a refresh rate of 240 Hz or higher is also recommended.

## Data Availability Statement

The raw data supporting the conclusions of this article will be made available by the authors, without undue reservation.

## Ethics Statement

The studies involving human participants were reviewed and approved by the Institutional Review Board of Xi’an Jiaotong University. The patients/participants provided their written informed consent to participate in this study.

## Author Contributions

CH and GX contributed to conception and design of the study. CH wrote the first draft of the manuscript and performed the statistical analysis. PT and XZ wrote sections of the manuscript. KZ, WY, YJ, and XC organized the database. All authors contributed to manuscript revision, read, and approved the submitted version.

## Conflict of Interest

The authors declare that the research was conducted in the absence of any commercial or financial relationships that could be construed as a potential conflict of interest.

## Publisher’s Note

All claims expressed in this article are solely those of the authors and do not necessarily represent those of their affiliated organizations, or those of the publisher, the editors and the reviewers. Any product that may be evaluated in this article, or claim that may be made by its manufacturer, is not guaranteed or endorsed by the publisher.

## Footnotes

1
https://www.tftcentral.co.uk/reviews/asus_rog_swift_pg258q.htm
